# Atomic structure of Mg-based metallic glass investigated with neutron diffraction, reverse Monte Carlo modeling and electron microscopy

**DOI:** 10.3762/bjnano.8.119

**Published:** 2017-05-31

**Authors:** Rafał Babilas, Dariusz Łukowiec, Laszlo Temleitner

**Affiliations:** 1 Institute of Engineering Materials and Biomaterials, Silesian University of Technology, Konarskiego 18a St., 44-100 Gliwice, Poland; 2 Institute for Solid State Physics and Optics, Wigner Research Centre for Physics, P.O. Box 49, H-1525 Budapest, Hungary

**Keywords:** electron microscopy, metallic glasses, neutron diffraction, reverse Monte Carlo modelling, short-range order

## Abstract

The structure of a multicomponent metallic glass, Mg_65_Cu_20_Y_10_Ni_5_, was investigated by the combined methods of neutron diffraction (ND), reverse Monte Carlo modeling (RMC) and high-resolution transmission electron microscopy (HRTEM). The RMC method, based on the results of ND measurements, was used to develop a realistic structure model of a quaternary alloy in a glassy state. The calculated model consists of a random packing structure of atoms in which some ordered regions can be indicated. The amorphous structure was also described by peak values of partial pair correlation functions and coordination numbers, which illustrated some types of cluster packing. The *N* = 9 clusters correspond to the tri-capped trigonal prisms, which are one of Bernal’s canonical clusters, and atomic clusters with *N* = 6 and *N* = 12 are suitable for octahedral and icosahedral atomic configurations. The nanocrystalline character of the alloy after annealing was also studied by HRTEM. The selected HRTEM images of the nanocrystalline regions were also processed by inverse Fourier transform analysis. The high-angle annular dark-ﬁeld (HAADF) technique was used to determine phase separation in the studied glass after heat treatment. The HAADF mode allows for the observation of randomly distributed, dark contrast regions of about 4–6 nm. The interplanar spacing identified for the orthorhombic Mg_2_Cu crystalline phase is similar to the value of the first coordination shell radius from the short-range order.

## Introduction

Magnesium-based metallic glasses are often described as the most sought after alloys given the increasing demand for light weight and low cost materials with good functional properties [1–3]. Many chemical compositions of metallic glasses based on Mg have been extensively reported in recent years [4–7], but among all the studied glassy materials, the Mg-TM-RE (TM – transition metal: Cu, Ni, Zn, Ag; RE – rare-earth transition metal: Y, Gd, Nd) system is one of the most highly investigated [8]. The good mechanical properties together with a large supercooled liquid region and high glass-forming ability with critical cooling rates below 10^3^ K/s make the Mg-TM-RE amorphous alloys attractive for a wide range of engineering applications [9]. However, most of Mg-based glasses are very brittle, which can limit the utility of these materials [10].

Nevertheless, the structural characterization of Mg-based metallic glasses are less described and studied. Some works on the structural characterization have been conducted by investigating the ternary Mg–Cu–Y glasses. Gao et al. [11] performed ab initio molecular dynamics simulations of the structural evolution of a Mg_65_Cu_25_Y_10_ alloy from liquid to glass state. Moreover, Laws et al. [12] provided an analysis of the dynamic crystallization in Mg_65_Cu_25_Y_10_ bulk metallic glass using transmission electron microscopy and atom probe tomography. Despite this, detailed information on the atomic configuration of the multicomponent Mg-based glassy alloys is not often reported.

This work aims at describing the structure of Mg_65_Cu_20_Y_10_Ni_5_ glass before and after annealing by experimental and modeling methods. The annealing step was conducted to achieve the formation of nanocrystals embedded in an amorphous matrix. The studied alloy is a modification of the chemical composition of a very popular glass (Mg_65_Cu_25_Y_10_) synthesized for the first time by Inoue et al. [13–14]. The samples achieved by copper mold casting exhibited bulk form with a diameter up to 4 mm. Ren et al. [15] studied the impact of Ni on the Mg_65_Cu_25−_*_x_*Y_10_Ni*_x_* (*x* = 0, 3, 6 atom %) alloy system due to the growth of crystalline phases and the formation of an amorphous structure. They also found that a small amount of Ni could improve the glass-forming ability of a Mg–Cu–Y alloy.

In this work reverse Monte Carlo (RMC) modeling was applied to neutron diffraction (ND) data in order to propose the atomic structural model and to determine the local atomic structure and indicate the regions of atomic clusters. The amorphous and nanocrystalline structure of a multicomponent alloy was also observed by high-resolution transmission electron microscopy (HRTEM) using specific techniques of structure observation.

## Experimental

The investigations were conducted on multicomponent Mg_65_Cu_20_Y_10_Ni_5_ (atom %) metallic glass. The samples were prepared in the form of ribbons with a thickness of 0.08 mm and a width of 10 mm by the melt spinning (MS) technique [16–18]. The master alloys as starting materials for MS casting were achieved by the induction melting of pure Mg, Cu, Y and Ni under an argon atmosphere. During the MS, the metallic liquid was rapidly quenched on the surface of a rotating copper wheel with a linear speed of 30 m/s. Moreover, the ejection over-pressure of the molten alloy under argon atmosphere reached a value of 0.03 MPa. The glassy samples were annealed at 473 K for 1 h to obtain nanocrystalline materials.

The amorphous materials can be also produced by other methods, including severe plastic deformation [19] or wet-chemistry deposition of thin films [20]. Severe plastic deformation leads to phase transitions and strong grain refinement in metallic alloys (e.g., Al–Zn, Al–Zn–Mg, Cu–Ni, Co–Cu, Ni–Y–Nb and Zr–Nb). It can result in the disordering of ordered phases, amorphization of crystalline phases and nanocrystallization in the amorphous matrix [19]. Straumal et al. [20] obtained nanometer scale grained oxide films by using the liquid ceramic technique (also called the wet-chemistry method). They obtained pure and doped ZnO films with thickness from 50 to 200 nm, which contained equiaxial grains of about 20 nm length. Moreover, the ZnO films exhibited ferromagnetic behavior.

The structure of the ribbons in the as-cast state was preliminary checked by conventional X-ray diffraction (XRD) in the reflection mode using a diffractometer with a Co Κα radiation source (wavelength λ = 0.179 nm). The diffraction patterns were collected by the step-scanning method in the 2Θ range from 30 to 90°.

The diffraction investigations were performed on the MTEST powder neutron diffractometer at the Budapest Neutron Center (BNC) in Hungary. The wavelength of the incident radiation was λ = 0.111 nm. The diffractometer is equipped with a monochromater changer that uses Ge(111), Cu(111) or Cu(220), allowing the beam to be adjusted for the required *Q*-range and resolution. The scattering intensity was measured up to the maximum scattering vector *Q*_max_ = 100 nm^−1^. The scattering vector *Q* is defined as the difference between the wave vectors, each with the magnitude 2π/λ, in the direction of the incident and scattered beam *Q* = 4πsinθ/λ, where 2θ is the angle between the incident and scattered beam and λ is the wavelength. The ribbons for ND measurements were first cut into small pieces and then placed inside thin-walled vanadium cans of 8 mm in diameter. The diffraction patterns of the empty and filled cans were recorded and then the intensity of the empty cans was subtracted from the total intensity. The intensities of the raw experimental data are normalized by a vanadium rod sample and background corrected. Then, previously measured datasets were corrected by the MCGR software [21] to remove the remaining systematical errors.

The ND data are represented by the structure factor *S*(*Q*) calculated as:

[1]
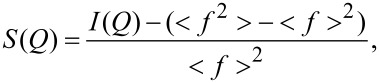


where 
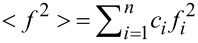
 and 
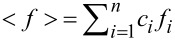
.

*I*(*Q*) is the corrected, normalized intensity, *c_i_* is the atomic concentration of the *i-*th element, *f_i_* is the atomic scattering factor of the *i-*th kind element, and *n* is the number of the atomic species in the specimen [22].

The glass transition (*T*_g_), onset (*T*_x_) and peak crystallization (*T*_p_) temperatures of the studied samples were determined by differential scanning calorimetry (DSC) in the temperature range from 350 to 600 K at a constant heating rate of 20 K/min under argon atmosphere.

The reverse Monte Carlo (RMC) calculations were carried out by fitting to the ND *S*(*Q*) function using the software package RMC_POT_i64_1.3.0 [23]. A starting configuration of 8,000 atoms with set composition, randomly distributed in a cube box of length of 5.54 nm, was used. The number atomic density ρ = 0.0470319 Å^−3^ was calculated from the chemical composition using macroscopic density determined by Archimedes method. The following cut-off distances were entered throughout the simulation runs: Mg–Mg, 0.32 nm; Mg–Cu, 0.29 nm; Mg–Y, 0.34 nm; Mg–Ni, 0.28 nm; Cu–Cu, 0.26 nm; Cu–Y, 0.31 nm; Cu–Ni, 0.25 nm, Y–Y, 0.36 nm; Y–Ni, 0.30 nm; Ni–Ni, 0.25 nm.

The partial pair distribution function, *g*(*r*), was calculated from the final RMC model according to [24]:

[2]
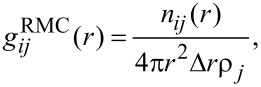


where *n_j_*(*r*) is the number of atoms of type *j* at a distance between *r* and *r +* Δ*r* from a central atom of type *i* and ρ*_j_* is the number density of atoms of the type *j*.

The calculated and the experimental data during the RMC simulation are compared by calculating the χ^2^ [23]:

[3]
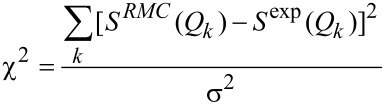


where *k* is intersects the points of the data set.

An amorphous structure with nanocrystals was observed using a high-resolution transmission electron microscope S/TEM TITAN 80-300 by FEI. The high-resolution transmission electron microscopy (HRTEM) images, selected area electron diffraction (SAED) patterns and energy dispersive spectroscopy (EDS) spectra were collected. Samples of nanocrystalline material for HRTEM observation were prepared by gallium ion milling.

## Results and Discussion

**Figure 1:**
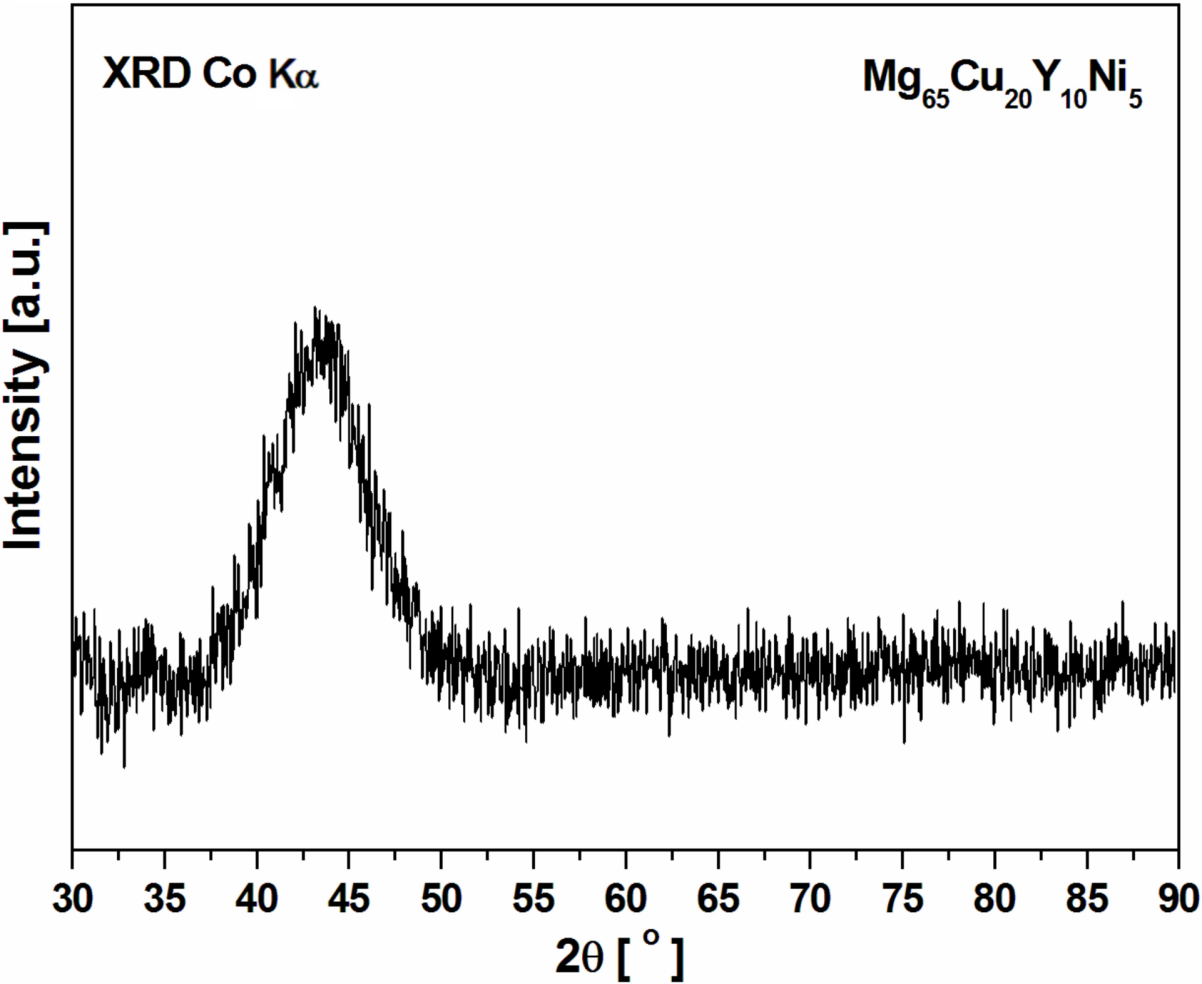
X-ray diffraction pattern of a Mg_65_Cu_20_Y_10_Ni_5_ glassy ribbon in the as-cast state.

Figure 1 shows the conventional XRD pattern obtained for the ribbon in the as-cast state. The XRD pattern consists only of a broad diffraction peak in the 2θ range of 35–50°. The broad diffraction maximum was centered at about 43° and indicated the formation of an amorphous phase. Moreover, the position of the broad maximum is known to be directly related with the average radius (*R*) of the first coordination shell. The *R* can be calculated by using following formula λ/2sinθ^*^, where 2θ^*^ is the scatter angle at the halo maximum and λ is the wavelength [25]. The value of *R* = 0.242 nm can be compared with the atomic radii of the Mg, Cu, Y and Ni elements, which is 0.160, 0.128, 0.180 and 0.124 nm, respectively [26]. It is noted that a value of the first coordination shell radius is similar to the interplanar spacing of 0.241 nm, which could be identified for the orthorhombic Mg_2_Cu crystalline phase.

In [27–28], synchrotron XRD was used to confirm the amorphous structure of as-cast Mg_60_Cu_30_Y_10_, Mg_65_Cu_20_Y_10_Zn_5_ and Mg_65_Cu_20_Y_10_Ni_5_ bulk alloys. The radius of the first coordination shell was determined to be 0.241 nm for Mg_60_Cu_30_Y_10_, 0.244 nm for Mg_65_Cu_20_Y_10_Zn_5_ and 0.243 nm for Mg_65_Cu_20_Y_10_Ni_5_ metallic glass. It is noted that the values of *R* obtained from the high-energy XRD patterns are very similar to those determined from the conventional XRD results.

Moreover, the value of *R* can be also related to the formation of icosahedral MgCu clusters. Jovari et al. [29] examined the short-range order of amorphous Mg–Cu–Y glass and also reported that Mg–Mg and Mg–Cu nearest atomic distances are very similar to values stated for crystalline Mg_2_Cu phase. The nanometer scale diffraction from selected areas in an amorphous matrix of Mg_60_Cu_30_Y_10_ glass used in [27] allows the interplanar spacing to be determined, which comes from the Mg_2_Cu clusters. What is more, the corrosion resistance of amorphous materials is related to the atomic structure. The lowest corrosion current density was detected for Mg_60_Cu_30_Y_10_ metallic glass, which has the lowest value of the first coordination shell radius from all *R* values determined for Mg_65_Cu_20_Y_10_Zn_5_ and Mg_65_Cu_20_Y_10_Ni_5_ alloys [28].

**Figure 2:**
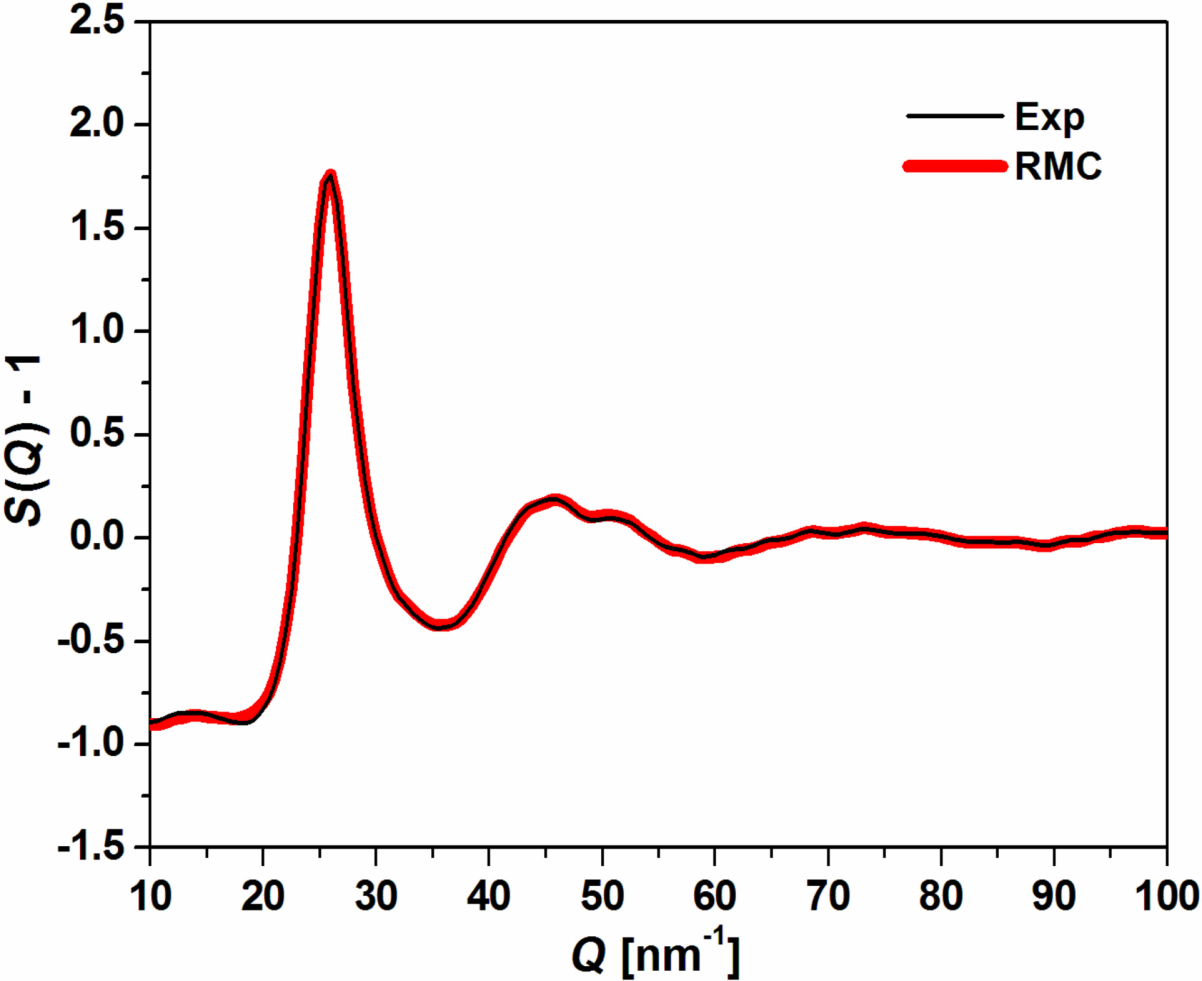
Experimental (black line) and reverse Monte Carlo modeling fit (red line) structure factors for Mg_65_Cu_20_Y_10_Ni_5_ metallic glass.

The ND structure factor *S*(*Q*) determined from experimental neutron diffraction data is shown in Figure 2. In order to achieve the topological model of the studied structure, RMC modeling based on the neutron diffraction data was used. Additionally, the *S*(*Q*) calculated from the RMC model is also shown in Figure 2 where it can be seen that both *S*(*Q*) values for *Q* in the range 10–100 nm^−1^ exhibited a very good quality fit. The calculated structure factor indicated that the final configuration of atoms for the amorphous Mg_65_Cu_20_Y_10_Ni_5_ alloy simulation was reasonable.

**Figure 3:**
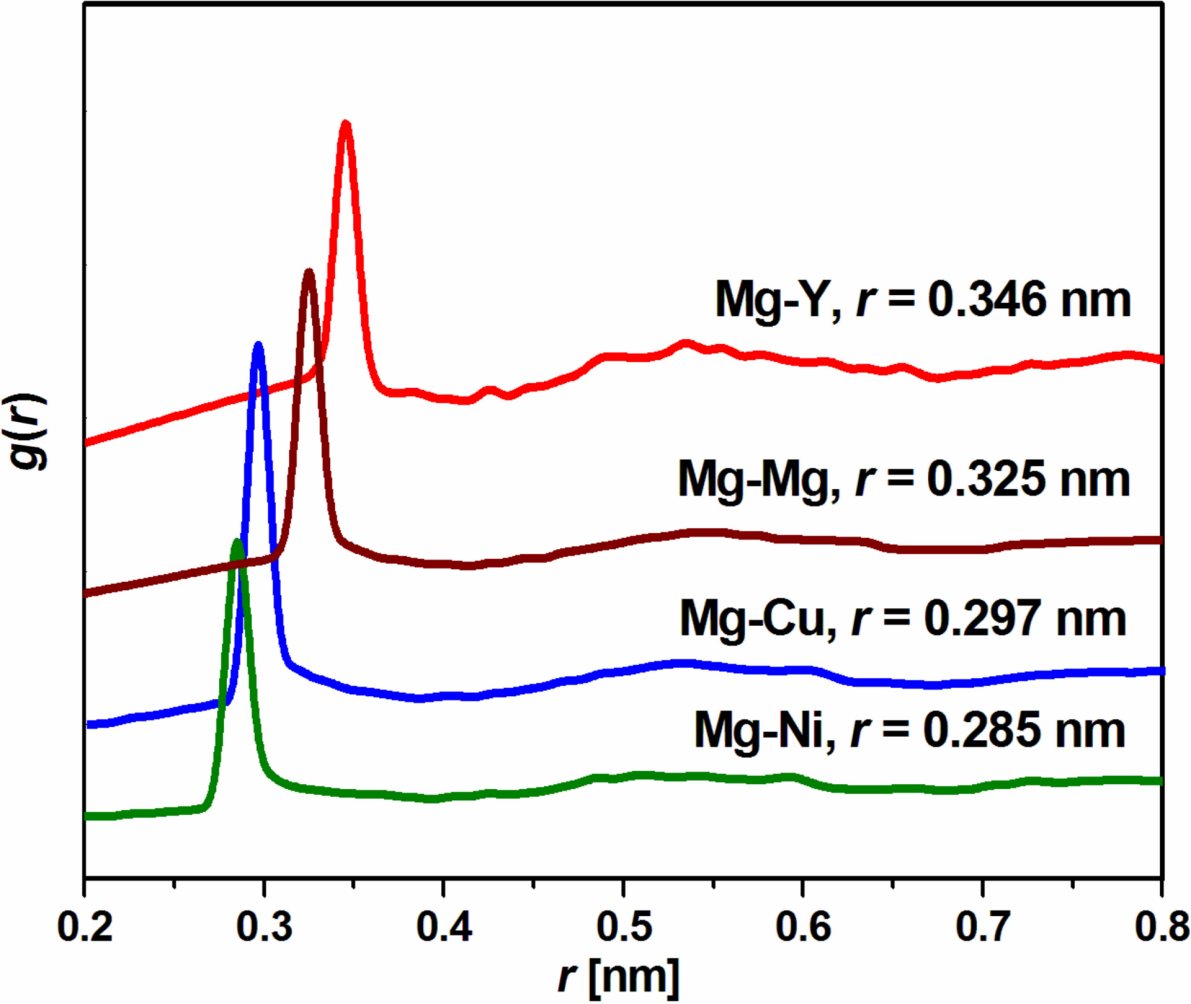
Partial pair distribution functions of Mg_65_Cu_20_Y_10_Ni_5_ metallic glass obtained from reverse Monte Carlo calculations.

Figure 3 presents partial pair distribution functions (PDFs), *g*(*r*), calculated from the final structural model of Mg_65_Cu_20_Y_10_Ni_5_ metallic glass. The PDF function is one of the main tools which are used to describe the local atomic structure in amorphous materials. The peak values of Mg–Y, Mg–Mg, Mg–Cu and Mg–Ni partial functions *g*(*r*) are determined at 0.346, 0.325, 0.297 and 0.285 nm. The calculated distance between atomic pairs is very close to the sum of the nominal radii of Mg–Mg or Mg–Y atoms.

The similar results of Mg–Mg, Mg–Cu and Mg–Y atomic pairs are reported by Jovari et al. [29] for the Mg_60_Cu_30_Y_10_ metallic glass in the bulk and thin ribbon form. The atomic structure of glassy samples was characterized by high-energy X-ray and neutron diffraction as well as the EXAFS method. The obtained experimental data were fitted by RMC modeling. Also, Gao et al. [11] used ab initio molecular dynamics to describe the atomic structure of a Mg_65_Cu_25_Y_10_ alloy during transformation from the liquid to glass state. The pair correlation functions, coordination numbers and structure factors were calculated.

**Figure 4:**
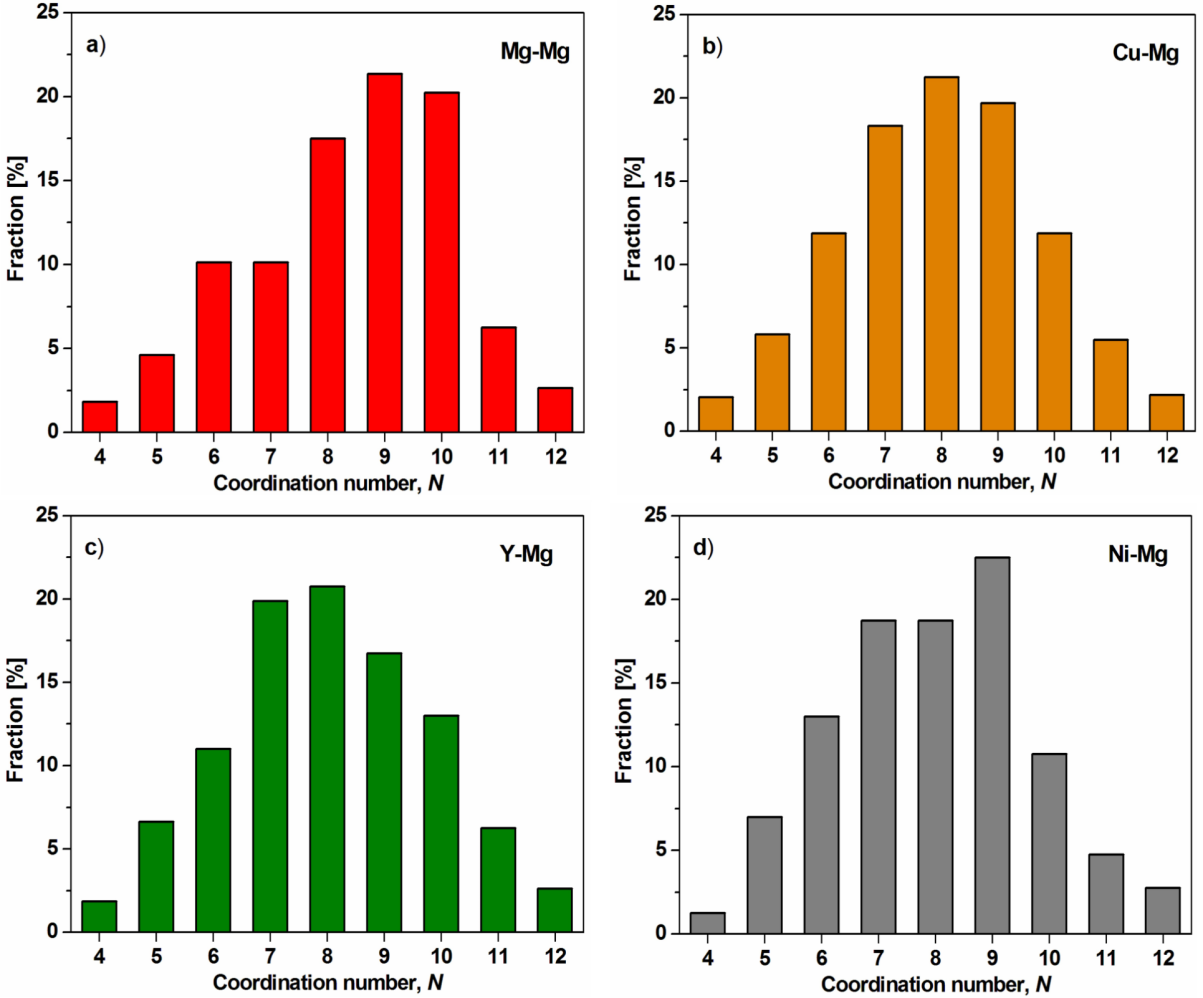
Distribution of the coordination number of Mg–Mg (a), Cu–Mg (b), Y–Mg (c) and Ni–Mg (d) atoms in Mg_65_Cu_20_Y_10_Ni_5_ metallic glass.

The distributions of Mg-, Cu-, Y- and Ni-centered coordination numbers are shown in Figure 4. The nearest-neighbor coordination number (*N*) presents the dominant coordination polyhedron and also describes the short-range order (SRO). It can be observed that the distributions of *N* around Mg and Cu atoms are quite similar. However, the *N* = 9 clusters are dominant around Mg atoms, but the *N* = 8 clusters have the highest fraction around Cu atoms. On the other hand, the *N* = 8 clusters exhibit the largest population around Y atoms and atomic clusters with *N* = 9 are also dominant for Ni–Mg atoms. These results should be caused by the different atomic radius of elements. The calculated values of inter-atomic distances and coordination numbers are also listed in Table 1.

**Table 1:** Inter-atomic distances (*r*) and coordination numbers (*N*) of Mg_65_Cu_20_Y_10_Ni_5_ glass.

Sample	*r* (nm)	*N*	Atomic pair

Mg_65_Cu_20_Y_10_Ni_5_	0.346 (± 0.02)	1.2 (± 0.2)	Mg–Y
	0.325 (± 0.02)	8.2 (± 0.5)	Mg–Mg
	0.297 (± 0.02)	2.5 (± 0.3)	Mg–Cu
	0.285 (± 0.02)	0.6 (± 0.1)	Mg–Ni

The *N* distributions illustrated many types of cluster packing. Among others, the *N* = 9 clusters correspond to the tri-capped trigonal prisms (TTPs), which are one of Bernal’s canonical clusters [30]. Miracle et al. [31] showed that atomic clusters with *N* = 6 and *N* = 12 are suitable for octahedral and icosahedral atomic configurations. Furthermore, for *N* = 10, several clusters with similar packing efficiencies can be possible. The population of TTP clusters is the highest for Ni–Mg atomic pairs. The population of *N* = 7 clusters around Y atoms is dominant in comparison with other coordination number distributions. Comparing with the distribution of *N* = 7 for Mg–Mg pairs, the distribution of the number of adjusted clusters is slightly different. Nevertheless, the obtain results could also lead to the conclusion that cluster packing in Mg_65_Cu_20_Y_10_Ni_5_ glass has a nine-fold coordination.

**Figure 5:**
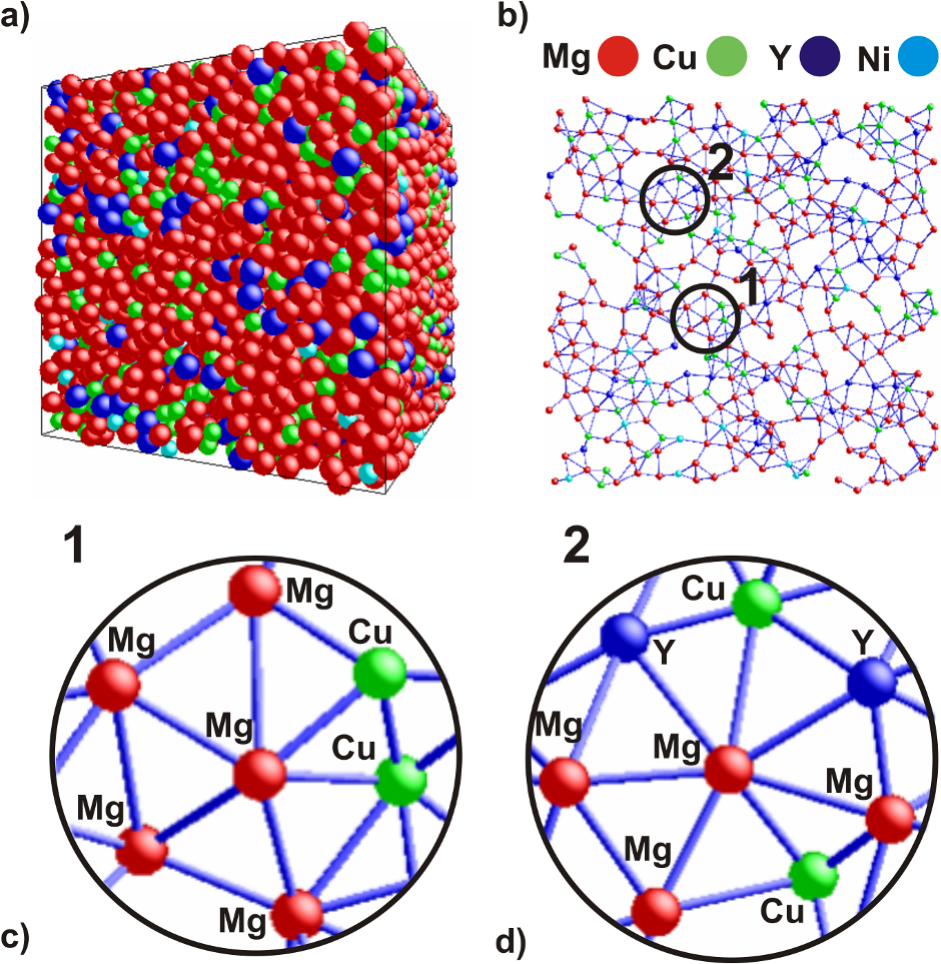
Structure of Mg_65_Cu_20_Y_10_Ni_5_ metallic glass determined by reverse Monte Carlo modeling: (a) simulation box, (b) a layer with thickness of 0.5 nm taken from the (100) plane and (c,d) indication that the SRO regions come probably from clusters.

Figure 5 presents the 3D atomic configuration obtained from RMC modeling of the random configuration of 8,000 atoms. The output box (Figure 5a) shows that the distribution of atoms is not completely homogeneous. It can be seen that the Cu and Y atoms segregate in some areas, indicating the formation of local ordering with more and less dense regions. Similar results of RMC modeling were obtained for a Zr-based amorphous alloy by Hui et al. [32]. The 3D atomic configuration of Zr_2_Ni metallic glass was inhomogeneous with local segregations of atoms. Moreover, a layer of 0.5 nm thickness was extracted from the RMC output box in order to visualize some atomic configurations (Figure 5b). The selected areas in the extracted atomic layer indicates that some types of SRO regions probably come from clusters. In selected SRO regions, the presence of hexagons (Figure 5c) and heptagons (Figure 5d) can be observed in the 2D view. The hexagons can be assumed as a representation of six-fold coordinated numbers, while the configuration shown in Figure 5d is characteristic for seven-fold coordination, which can also be found in the distribution of *N* exhibited in Figure 4.

**Figure 6:**
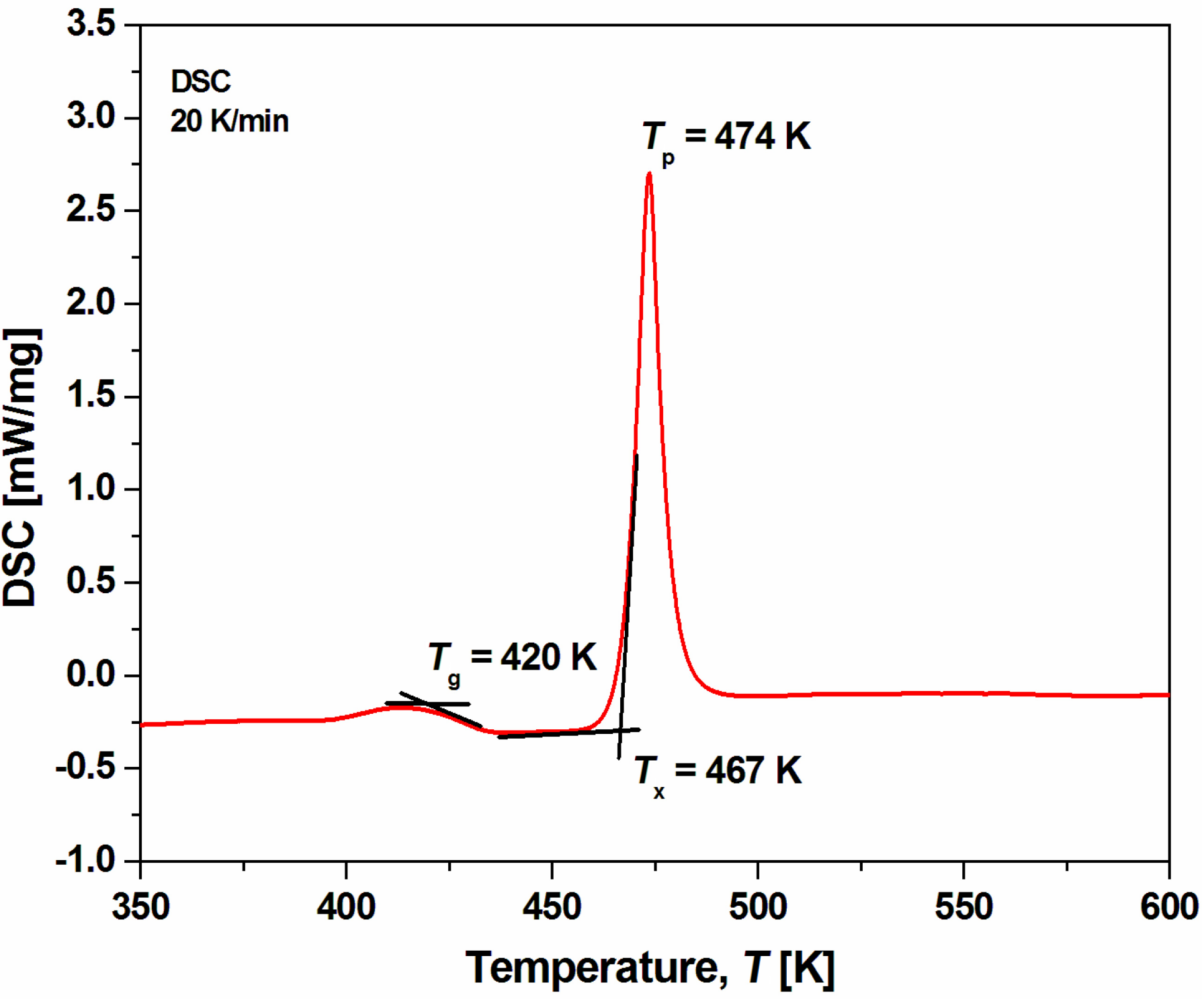
DSC curve of the Mg_65_Cu_20_Y_10_Ni_5_ ribbon sample in the as-cast state.

Figure 6 shows the DSC curve of the Mg_65_Cu_20_Y_10_Ni_5_ ribbon sample. The sample was heated from room temperature to 600 K at a heating rate of 20 K/min. It can be observed that the amorphous ribbon exhibited an endothermic effect of the glass transition followed by a distinct exothermic peak. The detected effects confirmed the amorphous structure of the studied sample and allowed the glass transition temperature (*T*_g_ = 420 K), the onset crystallization temperature (*T*_x_ = 467 K) and the peak crystallization temperature (*T*_p_ = 474 K) to be determined. The supercooled liquid region (Δ*T*_x_ = *T*_x_ − *T*_g_) is about 54 K. Compared to the Mg_65_Cu_20_Y_10_Zn_5_ glassy alloy described by Men et al. [33], the Mg_65_Cu_20_Y_10_Ni_5_ ribbon exhibits a higher onset crystallization temperature and higher Δ*T*_x_ parameter. The addition of nickel can improve the thermal stability of Mg-based metallic glasses. Moreover, the determination of the crystallization temperature is helpful to prepare nanocrystalline samples by heat treatment.

**Figure 7:**
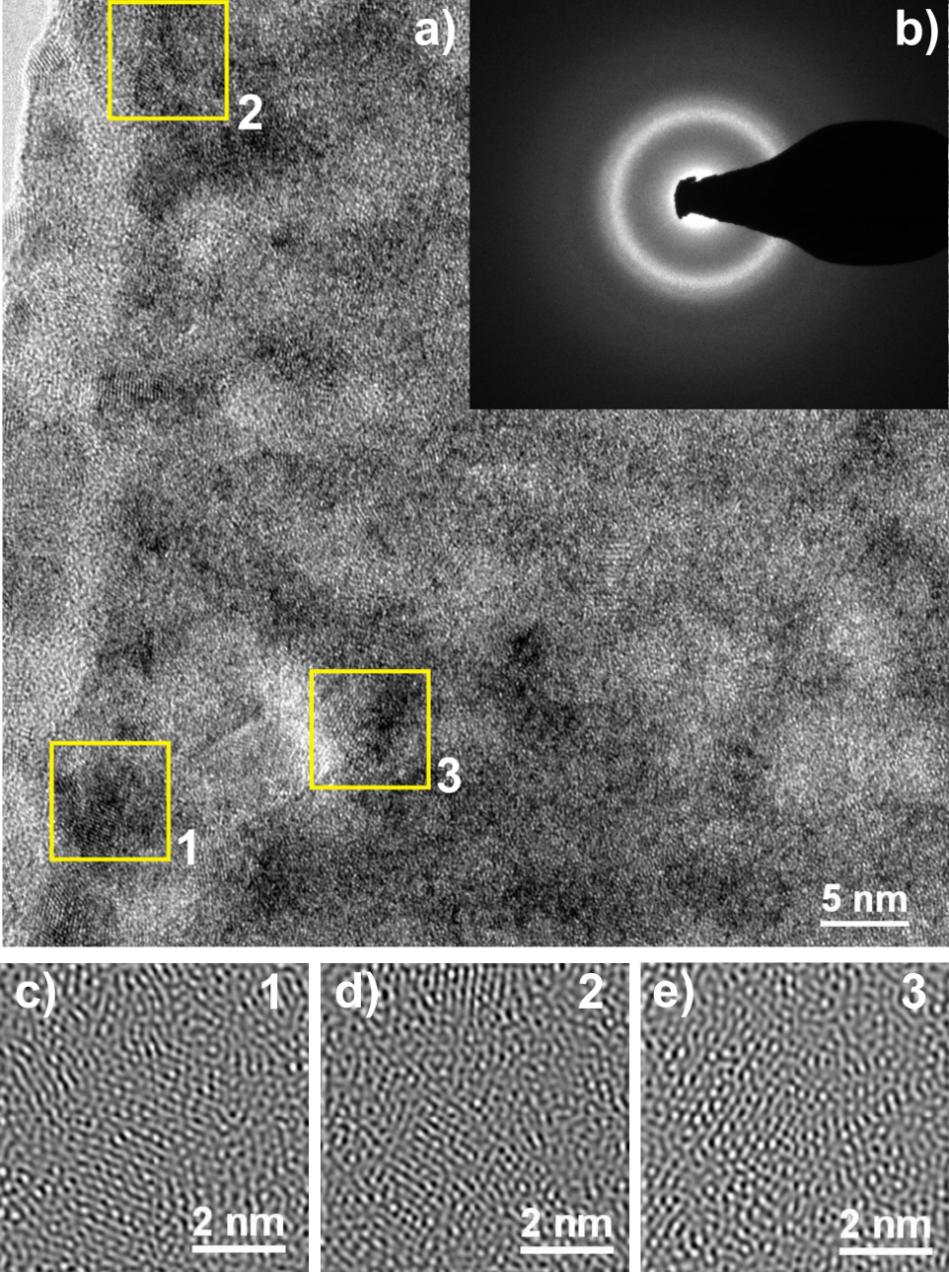
(a) HRTEM image, (b) selected area electron diffraction (SAED) pattern and details of the selected areas 1, 2, 3 (c,d,e) of a Mg_65_Cu_20_Y_10_Ni_5_ metallic glass after annealing at 473 K for 1 h.

Figure 7 presents a high-resolution transmission electron microscope (HRTEM) image and corresponding selected area electron diffraction (SAED) patterns of a sample that was annealed at 473 K for 1 h. The annealing temperature was selected based on the results of the DSC measurements. The SAED image (Figure 7b) consists of a halo pattern from the amorphous matrix and some rings from the ordered regions. A careful analysis of the HRTEM image (Figure 7b) reveals some periodic fringe contrast regions (Figure 7a). To observe more clear microscopic images of the ordered areas on the nanoscale, the selected HRTEM images of the nanocrystalline regions were processed by inverse Fourier transform (IFT) functions (Figure 7c–e). Small, ordered regions of about 6 nm can be seen. These regions were marked by squares numbered 1 to 3. Moreover, the identification of nanocrystalline structure was difficult, especially due to the broad halo patterns.

**Figure 8:**
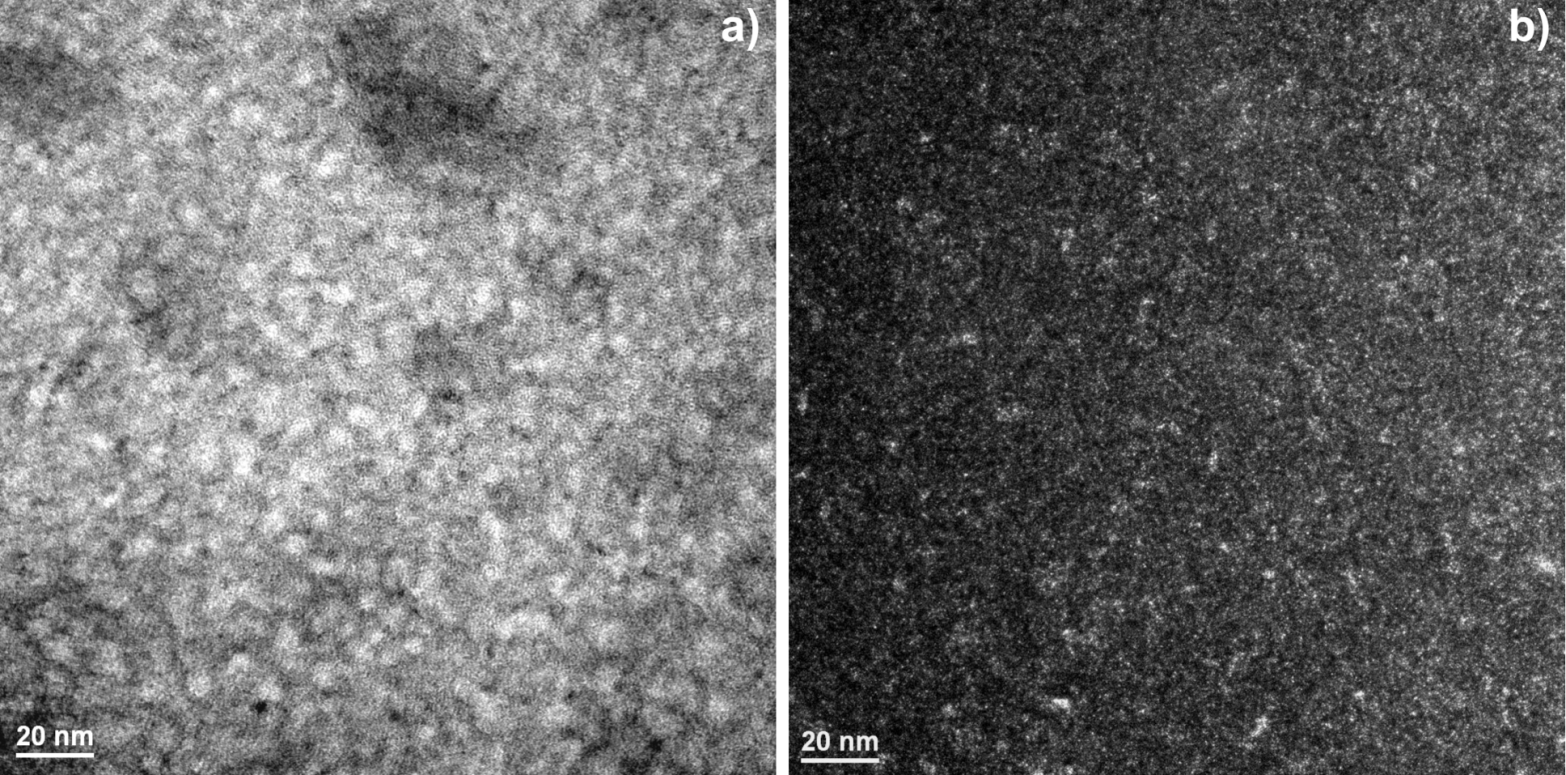
TEM images in (a) bright field and (b) dark field mode of a Mg_65_Cu_20_Y_10_Ni_5_ metallic glass sample after annealing at 473 K for 1 h.

The nanocrystalline behavior of the studied glass after annealing was also studied by conventional TEM in bright (Figure 8a) and dark (Figure 8b) field. The alloy in the as-annealed state exhibits a microstructure consisting of homogeneously dispersed nanometer-sized fine particles. The black and grey particles are dispersed randomly and densely in the amorphous matrix.

**Figure 9:**
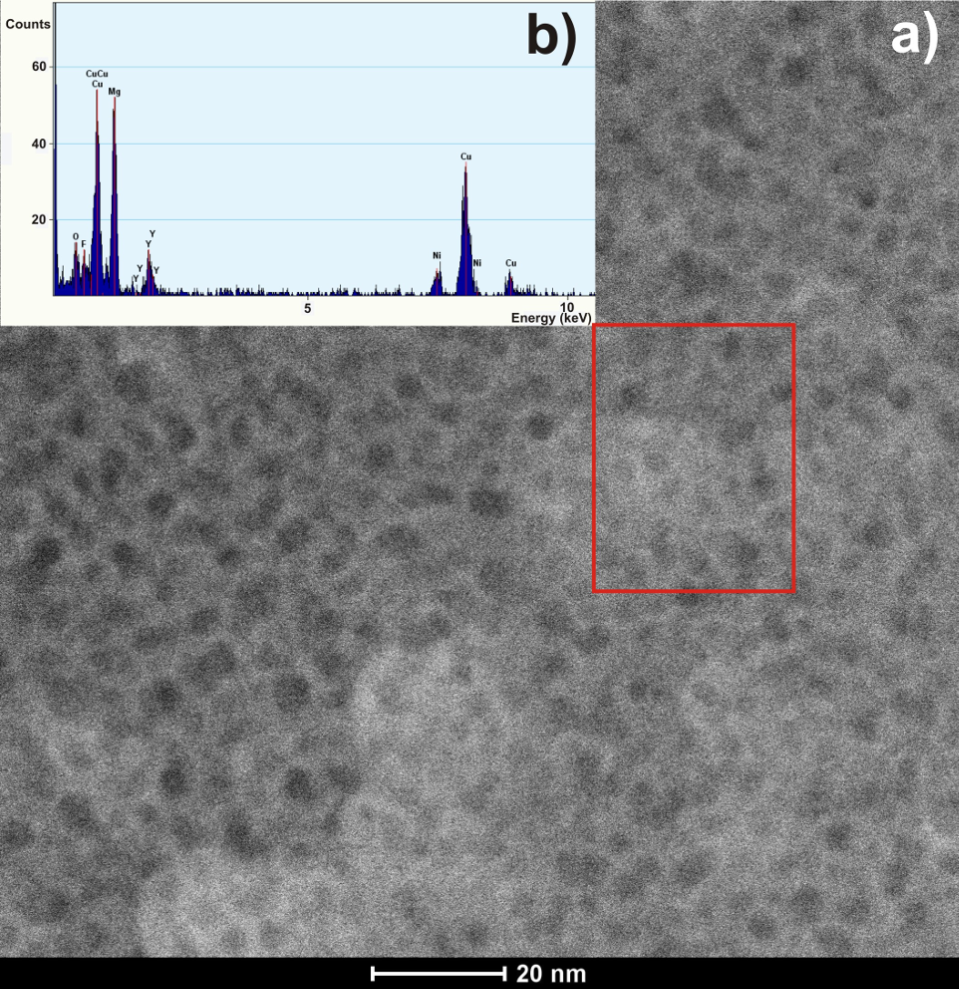
(a) HAADF-STEM image and (b) EDS spectrum from a selected area of the Mg_65_Cu_20_Y_10_Ni_5_ metallic glass after annealing at 473 K for 1 h.

The high-angle annular dark-ﬁeld (HAADF) results were used to observe phase separation in the studied glass after heat treatment. The image collected in HAADF mode allows randomly distributed, dark contrasts to be observed (Figure 9a). The area of the dark contrast regions calculated from the HAADF image is 4 × 6 nm. In addition, the EDS spectrum (Figure 9b) obtained in the HAADF-STEM mode confirmed the qualitative composition of the tested sample as the nanocrystalline material. Hirotsu et al. [34] used the HAADF technique to indicate a local compositional fluctuation caused by nanoscale phase separation in Zr–Cu–Ni–Al–Ti bulk metallic glasses. They achieved bright contrast regions with size above 2 nm. The authors also stated that the identified contrasts are in good agreement with observations conducted in high-resolution mode.

**Figure 10:**
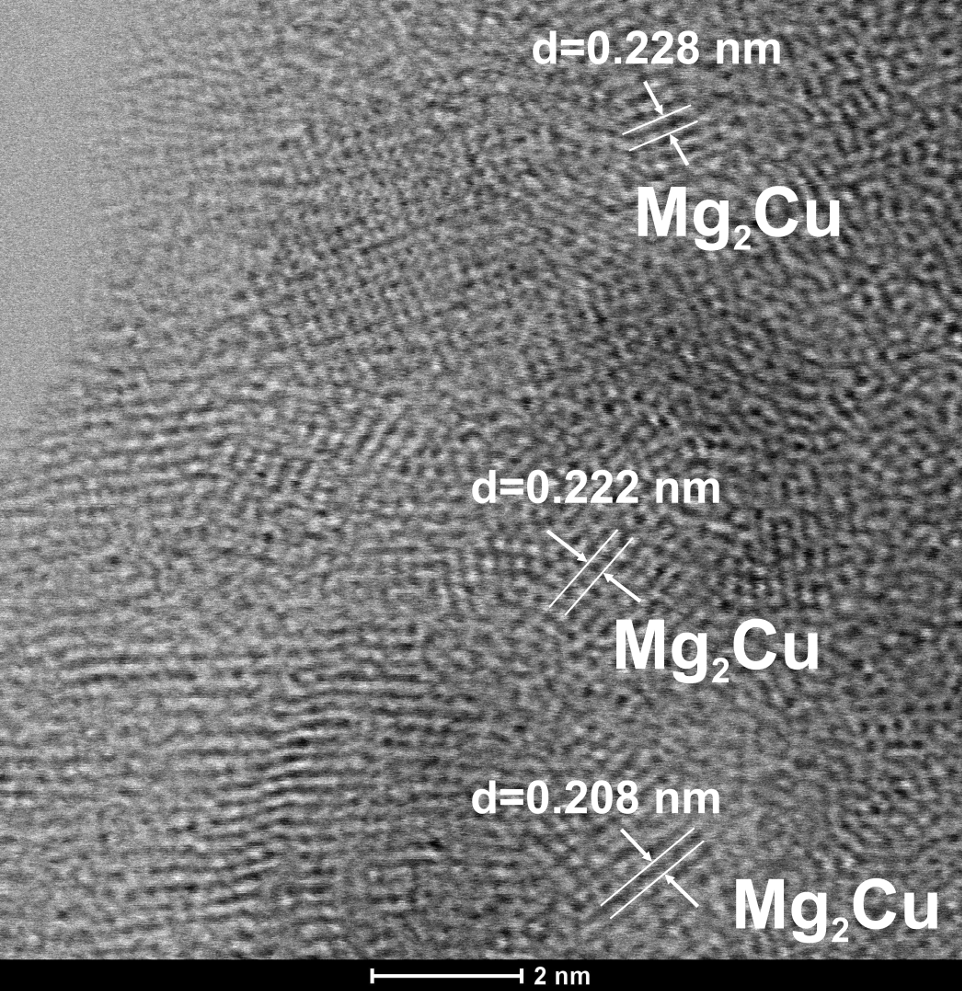
STEM-BF image of Mg_65_Cu_20_Y_10_Ni_5_ metallic glass after annealing at 473 K for 1 h.

Further analysis of the ordered areas formed in the specimen annealed at 473 K for 1 h was provided by using STEM mode. The STEM bright field image presents some areas with a crystalline structure that indicate the formation of a primarly crystallization phase (Figure 10). The interplanar spacings with values of *d* = 0.208 nm, *d* = 0.228 nm and *d* = 0.222 nm were found in the crystalline regions. The measured values of *d*-spacings seem to be closely correlated with the interplanar spacings of intermetallic Mg_2_Cu phase.

**Figure 11:**
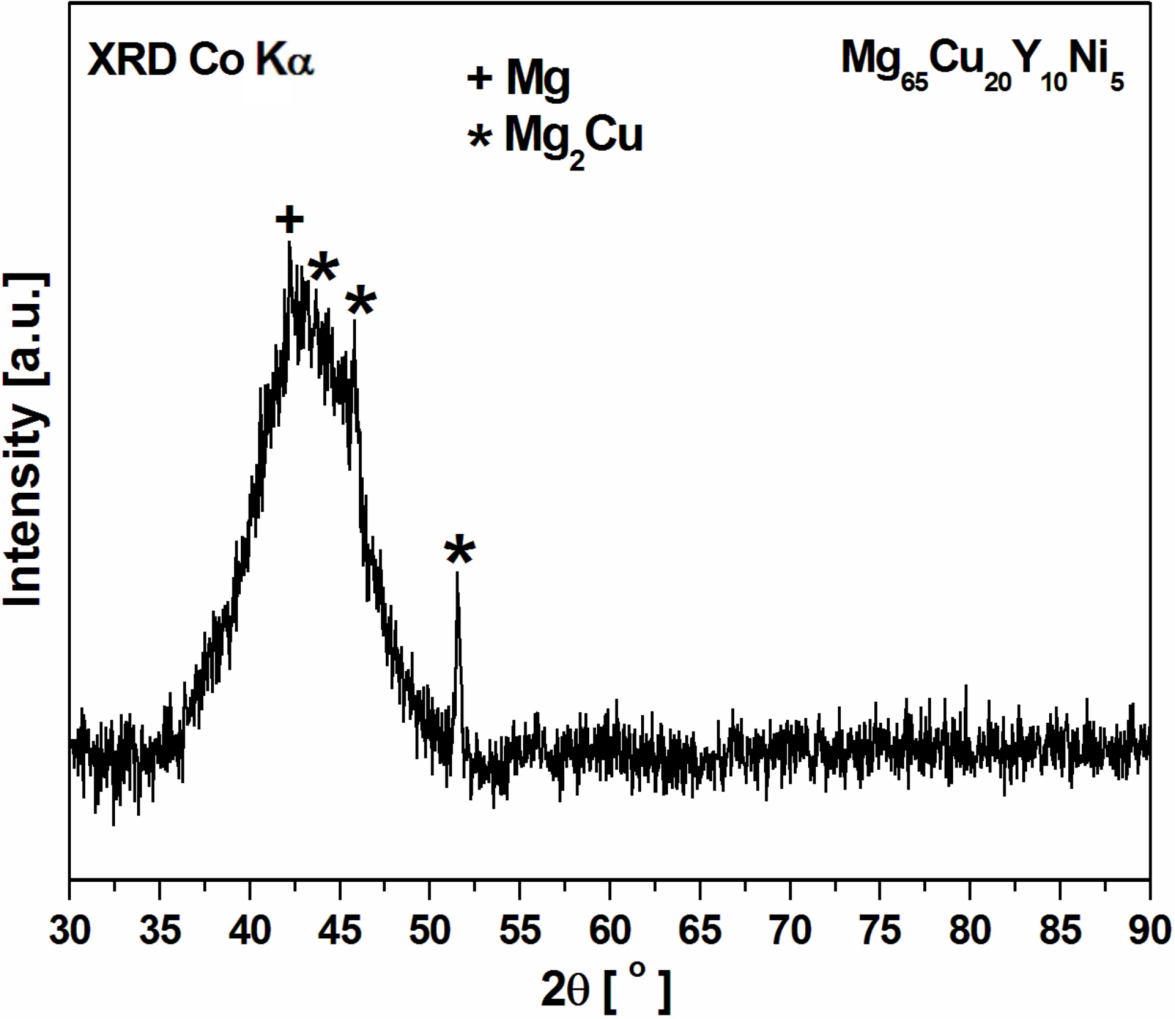
XRD pattern of Mg_65_Cu_20_Y_10_Ni_5_ alloy after annealing at 473 K for 1 h.

The XRD pattern (Figure 11) of the Mg_65_Cu_20_Y_10_Ni_5_ alloy after annealing at 473 K for 1 h exhibits peaks superimposed on a broad diffraction halo. The peaks are described as Mg with Mg_2_Cu phases, indicating that the alloys are the glassy composite. For comparison, Lee et al. [35] presented the XRD patterns with crystalline peaks of Mg, Mg_2_Cu and Mg_24_Y_5_ phases and an amorphous background for Mg_61_Y_15_Cu_24_ as-milled samples after annealing at 443 K for 30 min.

Moreover, the authors identified the Mg and Mg_2_Cu phase by the synchrotron diffraction of Mg_60_Cu_30_Y_10_ bulk metallic glass after annealing at 473 K for 1 h. The nanobeam electron diffraction (NBED) patterns determined from nanocrystals with a diameter of 150 nm confirmed the formation of the Mg_2_Cu structure [27].

A comparison of the structural analysis results for Mg_60_Cu_30_Y_10_ [27] and Mg_65_Cu_20_Y_10_Ni_5_ metallic glasses in the as-cast state confirmed that the atomic model determined from RMC calculations represents some types of the SRO regions. The SRO areas probably come from atomic clusters. The selected SRO regions in the 2D view of the RMC model can be represented by hexagons and heptagons for Mg_65_Cu_20_Y_10_Ni_5_ glass and some pentagons and hexagons for Mg_60_Cu_30_Y_10_. The XRD investigations for both glassy alloys suggest a formation of Mg–Cu clusters in the first coordination shell. The nanocrystalline structure was also confirmed for the studied alloys after the annealing process and under the same conditions. However, it is important to note that larger spherical nanocrystals (≈150 nm in diameter) were observed in the postannealed structure of the Mg_60_Cu_30_Y_10_ alloy as compared to the Mg_65_Cu_20_Y_10_Ni_5_ annealed sample (smaller crystals with a diameter of less than 10 nm).

## Conclusion

In this paper, a set of advanced experimental methods, including the neutron diffraction, reverse Monte Carlo modeling and high-resolution electron microscopy were used to characterize the atomic structure of multicomponent, Mg_65_Cu_20_Y_10_Ni_5_ alloy in a glassy and postannealed state. A combination of the methods was very useful to describe the local atomic structure of the studied alloy in the glassy state and after structural transformation formed by heat treatment. The short-range order was described by distributions of the nearest-neighbor coordination number, which revealed the dominant coordination polyhedron. The coordination number distributions revealed that many types of cluster packing are possible. The *N* = 9 clusters should be related to the tri-capped trigonal prisms. Moreover, atomic clusters with *N* = 6 and *N* = 12 are suitable for octahedral and icosahedral atomic configurations. The nanocrystalline behavior of the studied glass after annealing was also studied by conventional and high-resolution TEM. The high-angle annular dark-ﬁeld (HAADF) observation was used to observe phase separation. The HAADF mode allowed randomly distributed, dark contrasts regions with size from 4 to 6 nm to be observed. The value of the first coordination shell radius was found to be similar to the interplanar spacings identified for orthorhombic Mg_2_Cu in the crystalline phase, which was identified during microscopy observations.
